# A Retrospective Analysis of Concordance Between Clinical and Histopathologic Diagnoses and Completeness of Oral Biopsy Forms at a Tertiary Dental Hospital in Eastern Nepal

**DOI:** 10.1155/2024/2528353

**Published:** 2024-10-03

**Authors:** Shashi Keshwar, Neetu Jain, Toniya Raut, Vimmi Singh, Ashish Shrestha

**Affiliations:** ^1^ Department of Oral Pathology College of Dental Surgery B.P. Koirala Institute of Health Sciences, Dharan, Nepal; ^2^ Department of Conservative Dentistry College of Dental Surgery B.P. Koirala Institute of Health Sciences, Dharan, Nepal

## Abstract

**Introduction:** Histopathological diagnosis remains the gold standard tool for the diagnosis, yet accurate and detailed clinical descriptions are necessary to facilitate the final diagnosis. Histopathologists believe that clinicians are unaware of how histopathology departments operate, partly because of the less information on requisition forms. The objective of the present study is to assess the concordance of clinical and histological diagnoses of all oral and maxillofacial biopsy samples, along with the completion of the requisition form provided with the biopsy sample.

**Methods:** A retrospective study was conducted at the Department of Oral Pathology. The biopsy request forms of year 2018–2019 were retrieved from the archive of the department and were analyzed for clinicopathological concordance. Descriptive and analytical statistics were performed using SPSS.

**Results:** Of 338 forms, 243 (71.89%) forms had total concordance between clinical and histopathologic diagnosis; 20 forms (5.92%) had concordance with the histopathological diagnosis, but only after the clinical diagnosis had been refined. Of all the forms analyzed, 36 (10.65%) forms lack habit history in cases suspected of oral cancer and oral potentially malignant disorder, and 24 (7.10%) cases lack radiographic details. The categories of clinicohistopathological concordance and the different clinical information groups showed a statistically significant relationship. We also found that the sign-out time for histopathological reports depend on the extent of clinical information provided which was statistically significant too.

**Conclusions:** The current study concluded a sufficient level of concordance between clinical and histopathological diagnosis. A high completion rate of biopsy forms indicated that the clinicians/operating surgeons perceive the significance of clinical information in histopathological diagnosis. We also recommend, irrespective of the type of suspected oral lesions, submitted for biopsy, a detailed clinical information is the backbone for accurate and timely reporting of the histopathological diagnosis.

## 1. Introduction

Clinical information (CI) and diagnostic tests usually have a major impact on the histopathological analysis of biopsied specimens, which is a crucial diagnostic tool [1, 2]. Histopathological diagnoses, a gold standard for diagnosing oral mucosal lesions, usually differ in clinical judgments [2–5]. Numerous studies have demonstrated that the histopathological interpretation is influenced by multiple factors that ultimately impact the diagnosis [6]. These disparities stem, in part, from methodological variations in diagnostic criteria and the evaluating professional. The pathologist's diagnosis-making process is inevitably subjective. The final “sign out” diagnosis depends on a variety of factors, that includes, the clinical aspects of the lesion, the surgeon's clinical impression, and the pathologist's experience and training [3, 7]. Although histopathologists are supposed to help clinicians to reach out the most accurate possible diagnosis, it is the responsibility of the clinicians to provide them with adequate and relevant CI [7]. Therefore, it is critical to note that the patient's clinical history needs to be accurately filled, to rule out the presence of any habits or systemic conditions that might be associated with the presented oral lesions [4].

According to Ferrara et al. [8], providing relevant CI to the histopathologist can change the initial diagnosis. Therefore, inadequate information or incompletely filled forms have a significant impact on the quality of the report and might also lead to the waste of valuable laboratory time [9]. Differentiation between grades is mandatory in the diagnosis of oral epithelial dysplasia. However, the inclusion of CI and the clinical description often changes the picture [10].

The clinical aspects of each oral lesion contribute to its diagnosis, whereas histopathological evaluation gives the definitive diagnosis in most cases. A histopathologist cannot examine the specimen as per the clinician's presumption until provided with clinical details, preliminary diagnosis, and the reason for conducting the biopsy. Moreover, inadequate CI results in delayed reporting and an erroneous diagnosis. As a result, the histopathological report does not meet the expectations of the clinician and does not answer the intended question [11].

Considering all these aspects, the entire process of diagnosis is complex and difficult, which varies depending on the type of oral disease. The existing literature shows inadequate information on the level of the completeness of the oral biopsy form and the concordance of the clinical and histopathological diagnosis of oral mucosal lesions in Nepal. Hence, the current study aimed to determine the level of completeness of biopsy request forms, as well as the percentage of concordance between clinical and histological diagnoses.

## 2. Materials and Methods

A retrospective observational study was conducted in the Department of Oral Pathology, College of Dental Surgery, B.P. Koirala Institute of Health Sciences, after obtaining ethical approval from the Institutional Review Committee (IRC/2086/020).

### 2.1. Sample Size Calculation

Soyele et al. [12] reported that absolute concordance was recorded to be 54.6% between clinical and histopathological diagnosis.

*Z* = 1.96, CI = 95%, and power = 80%.

Prevalence (*p*)=54.6% and compliment of prevalence (*q*) = 100 − *P*=45.4%.

Permissible error (*L*) = 10% of *P*=5.46.

Sample size (*n*) = *Z*^2^ × *p* × *q*/*L*^2^ = 307.

Adding 10% to reduce various biases, the final sample size = 338.

The biopsy request forms (2018–2019) were retrieved from the archive of the department, and information on clinical details and histopathologic diagnosis was collected. All the forms were classified into four groups based on the study conducted by Patel et al. [13] and were modified according to the needs of our study:

i. Total concordance (TC)—If there is TC between clinical and histopathological diagnosis.
ii. Concordance with histopathologic diagnosis but after refinement of clinical diagnosis (CARCD), for example, clinical diagnosis of leukoplakia with histopathological diagnosis of mild, moderate, or severe dysplasia.
iii. Disconcordance (DC)—If there is no concordance between clinical and histopathological diagnosis.
iv. No clinical diagnoses (NCD)—The clinician had failed to mention clinical diagnosis.

Data from the biopsy request form regarding CI were also segregated into three groups: long and detailed (LD), short and focused (SF), and deficient (D), according to the study done by Ali et al. [14] and modified according to the needs of our study (Table 1).

All the findings were entered into a spreadsheet and analyzed using SPSS, Version 23.0.

## 3. Results

Of the 338 forms assessed, 185 (54.73%) were of males and 152 (44.97%) were of females. All the requisition forms had mentioned the age of the patient, 330 (97.63%) forms had the patient's address, and 300 (88.75%) forms had mentioned the receiving date of the sample (Table 2).

**Figure 1:**
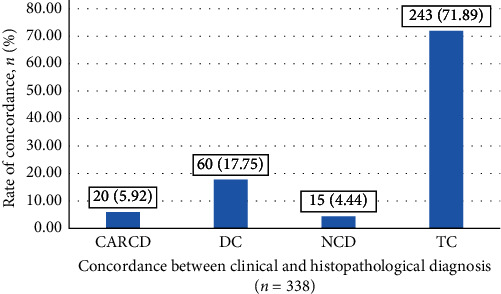
Concordance between clinical and histopathological diagnosis.

Among all the forms, TC between clinical and histopathological diagnosis was obtained in 243 (71.89%) forms. In total, 20 (5.92%) forms showed CARCD. DC was seen in 60 (17.75%) forms, and in 15 (4.44%) forms, there was NCD (Figure 1).

**Figure 2:**
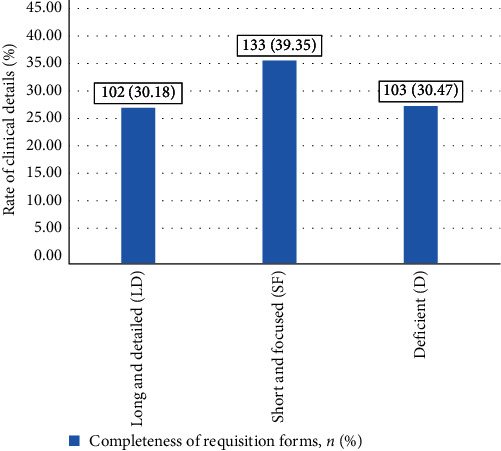
Completeness of requisition form.

Most of the forms 133 (39.35%) were found to have SF CI (Figure 2).

The various groups of concordance among clinical and histopathological diagnosis were compared with the various groups of CI, which showed that the percentage of concordance increases with the availability of short, focused, LD history in the requisition form, and the observation was statistically significant (Table 3).

**Figure 3:**
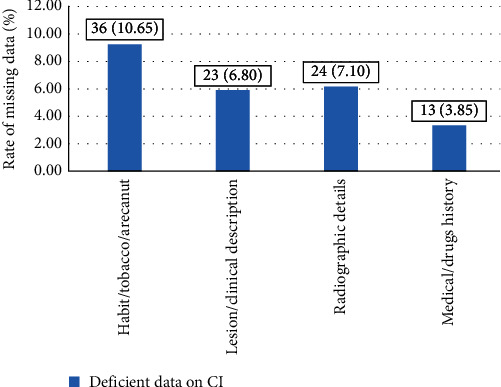
Deficient data on the clinical history.

Among all the forms analyzed, 36 (10.65%) forms in the case of oral potentially malignant disorders (OPMDs) and oral cancer diagnosis did not contain the history of the habit, and 24 (7.1%) forms did not have radiographic details (Figure 3).

The total forms were segregated based on the duration of sign-out time in days that showed maximum reports (50.59%) were dispatched within 5–7 days included those forms that primarily had a short, focused, and long detailed history. The cases that were dispatched within 8–10 days and more than 10 days were almost equal. The result also showed that only two (0.59%) forms with a LD CI took more than 10 days to be dispatched. The relation between clinical history detail and duration of sign-out time for the reports was statistically significant (Table 4).

## 4. Discussion

Oral lesions often present with identical clinical symptoms, making the diagnosis challenging for the clinician. Histopathological examination thereby becomes the way to an accurate diagnosis. However, accurate histopathological reports require some major information like age, sex, clinical history, clinical features, radiographic findings, and laboratory findings (biochemical, hematological, and microbiological parameters), if any. Hence, it is critical to have CI to make a firm diagnosis and develop an appropriate treatment plan.

The present study showed that the biopsy request forms received from the clinicians are adequately completed, although there is room for improvement. The demographic information and specifics about the specimen were provided in the majority of the forms, yet the patient's name and age were the only information that were included in 100% of the forms. The observation was in line with the findings of Sehgal et al. [9]. Abbasi, Asghari, and Niazkhani [15] found that name and age were documented in 99.8% and 91.96% of the forms, respectively. In contrast to our study, Adegoke et al. [16] found only 86.4% of the form included the age of the patient. Similarly, 97.63% of forms mentioned the address of the patient, which was in line with the study conducted by Burton and Stephenson [17] where it was 98.8%. The date and time of specimen collection were reported in 88.75% and 92.3% of forms, respectively. The documentation on time was only among 13.7% of the forms in the study conducted by Burton and Stephenson [17]. The department name was mentioned in 281 (83.13%) forms, which was in contrast to the study conducted by Sehgal et al. [9], where it was 100%. The consultant's name was written in 94.67% of the forms, which was in line with the study conducted by Abbasi, Asghari, and Niazkhani [15], where it was 98.68%. This contrasted with the study of Burton and Stephenson [17] and Sehgal et al. [9], where the figures were 47.5% and 62.4%, respectively.

Inadequate CI, including the details of operating surgeons, creates redundancy in the entire system of diagnosis. Proper use of histopathological services is an effective way of concluding the diagnosis, thereby preventing redundancy. The demographic information of the patient helps to identify them and enables comparison with earlier investigations and diagnoses. Identification errors can be prevented with the use of a written description of the nature of the sample. The complete name of the patient facilitates extracting information on the medical history of the patient from the health information system [18]. Hence, every single piece of information on the biopsy request form acts as an adjunct to each other that contributes individually or in conjunction with the accurate and timely diagnosis of the cases. The current study showed a total clinicopathological concordance rate of 71.89%, which was comparable to the results of studies conducted by Farzinnia et al. [2] and Saravani et al. [19]. However, our results contrast with the study done in Nepal, where the TC rate was 56.5% [20]. The discordance rate was 17.75%, which was in contrast with the studies conducted by Patel et al. [13], Poudel et al. [20], and Tatli et al. [21].

In our study, two-fifths (39.35%) of the samples had SF CI, which was in line with the study conducted by Ali et al. [14], where it was 38%. Nakhleh, Gephardt, and Zarbo [7] found that 2.4% of histopathology samples from 417 US laboratories lacked CI. However, in our study, D CI was observed in 30.47% of the total samples, which was in line with the samples reviewed by Sharif et al. [18] in a Pakistan laboratory.

These contradictory results might have many reasons. In the first instance, a clinician treating simple cases, such as specimens of pyogenic granuloma and fibroma, will typically offer a brief but targeted CI. The clinician usually provides a long and comprehensive CI that covers every facet of the clinical situation for complicated cases such as malignant neoplasms and odontogenic tumors. Despite the fact this is not always the case, we have encountered complicated specimens with inadequate CI that do not meet the pathologist's standards.

The group of CI in our study showed a statistically significant association with the group of concordance and highlighted that discordance is mainly seen in cases with D clinical details. The finding is strongly supported by studies that recommend interaction between clinicians and reporting pathologists to enhance the accuracy of histopathological diagnosis [3, 13, 15].

Most of the specimens with extensive CI were from the oral surgery department of the organization. It implies that the recommending clinicians from other departments of the hospital were a little clumsy. In our settings, filling out the request forms and giving them to patients or their attendees is often the responsibility of the subordinate house officers or junior residents. Sadly, they lack this kind of training and are frequently left on their own. On receiving such samples with inadequate CI, our pathologists routinely attempt to get in touch with the referring clinician/surgeons and obtain CI as quickly as possible.

The data regarding habit history (tobacco or tobacco products) were missing in one-tenth (10.65%) of requisition forms. Habit history is crucial for OPMD and oral cancer cases. Similarly, radiological data were missing in 7.1% of the forms. The significance of clinical data increases with radiological findings, which together solve many quires arise during histopathological slide interpretation. Data on the description of the lesion were missing in 6.8% of the forms, which was in contrast to the study conducted by Abbasi, Asghari, and Niazkhani [15], where it was 87.95%. The medical or drug history was missing in 3.85% of the forms. To identify drug-related pathology or possible changes in clinical symptoms as a result of drug therapy, precise medication histories are helpful [22]. Drug history is important in the situation where oral lichenoid lesions (OLLs) are to be differentiated from oral lichen planus (OLP). One significant distinction between OLL and OLP, despite the fact that these two lesions share a common clinical presentation in many cases, is that OLL has a known etiological agents, such as medicine [23, 24].

The study by Nakhleh and Zarbo [25] found that the most frequently obtained deficit information was “no clinical history or diagnosis present on the requisition slip,” accounting for 40% of all deficits, with a 77% rate of errors of discrepant or missing information elements. Few other studies showed the missing rates for the clinical history ranged from 50% to 85.3% [11, 26].

Histopathological examinations are mostly indicated in cases where clinical and radiographic evaluations are not confirmatory. Therefore, histopathology becomes the most awaited result for further treatment plans and patient management. A quality histopathological report refers not only to the diagnostic accuracy but also to the “timely” reporting of the accurate diagnosis [27]. In our study, the minimum turnover time/sign-out time of the report was 5–7 days. Considerable cases dispatched during this sign-out time had either SF or LD histories. Moreover, the majority of the cases that took more than 10 days' time to dispatch had D clinical details. The study conducted by Ali et al. [14] concluded that the SF CI was associated with a shorter turnaround time for the dispatch of the report. Abbasi, Asghari, and Niazkhani [15] also emphasized that the retrieval of information from the clinician results in the prolongation of the sign-out time for the diagnosis. Comprehensive data collected by clinicians/operating surgeons play a crucial role in the diagnostic procedure. Missing CI might be subjected to delayed reporting or redundancy, thereby compromising the prognosis and outcome of the disease [17].

Since ours is a teaching hospital, it is logical, that the dental professionals signing the request forms understand how important it is to complete the forms accurately; that is why most of the forms have adequacy for CI that helped in establishing histopathological diagnosis.

## 5. Conclusions

The present study establishes an adequate degree of concordance between the clinical diagnosis and the histopathological diagnosis. The high percentage of completed biopsy forms suggests that the clinicians perceive the significance of CI in histopathological diagnosis. These results also highlight that clinicians and operating surgeons should duly complete the biopsy request form by themselves or under self-supervision to get an accurate diagnosis. We also recommend that, irrespective of the type of suspected oral lesions, submitted for biopsy, a detailed CI is the backbone for accurate and timely reporting of the histopathological diagnosis.

### 5.1. Limitations

The current study had certain limitations, as it lacks official differentiation between the specializations or occupations of the requesting clinicians. This study is limited by its cross-sectional design and is a single hospital-based study. Long-term studies with a larger sample size including both public and private hospitals should be carried out before the findings can be widely utilized.

## Figures and Tables

**Table 1:** Operational definition of clinical information groups.

Clinical information (CI) groups	Operational definitions
Long and detailed (LD)	Complete CI, including age, sex, habit history, radiographic data, patient's previous clinical history and/or comorbidities, biopsy site, and provisional diagnosis

Short and focused (SF)	One or more of the above criteria were missing, but the CI was sufficient for the pathologist to make a diagnosis and complete the report

Deficient (D)	Absence of CI or (if provided) some of the above criteria were missing. Pathologists had to seek more CI from the requesting clinician/laboratory to establish a diagnosis and complete the report

**Table 2:** The information provided in the histopathology request form by the clinicians (*n* = 338).

Patient identifiers		
Variables	*n*	Percentage
Gender		
Male	185	54.73
Female	152	44.97
Not mentioned	1	0.29
Age		
Present	338	100
Absent	0	0
Name		
Present	338	100
Absent	0	0
Address		
Present	330	97.63
Absent	8	2.36
Specimen-associated items		
Sample receiving date		
Present	300	88.75
Absent	38	11.24
Sample receiving time		
Present	312	92.30
Absent	26	7.69
Clinician identifiers		
Name of the consultant in charge		
Present	320	94.67
Absent	18	5.32
Department/clinical unit		
Present	281	83.13
Absent	57	16.86

**Table 3:** Concordance based on clinical details.

Concordance	History (*N* = 338)			*p*-Value
	Deficient, *n* (%)	Short focused, *n* (%)	Long detailed, *n* (%)	
DC	28 (8.28)	24 (7.10)	8 (2.36)	<0.001 ^*∗*^
TC	54 (15.97)	100 (29.58)	89 (26.33)	
CACRD	7 (2.07)	8 (2.36)	5 (1.47)	
NCD	14 (4.14)	1 (0.29)	0	

Abbreviations: CARCD, concordance with histopathologic diagnosis but after refinement of clinical diagnosis; DC, discordance; NCD, no clinical diagnosis; TC, total concordance.

^*∗*^Chi-square test.

**Table 4:** Case distribution according to the report sign-out duration based on history details.

Duration of the report sign-out time (days)	Total case, *n* (%)	History			*p*-Value
		Deficient, *n* (%)	Short and focused, *n* (%)	Long and detail, *n* (%)	
5–7	171 (50.59)	8 (2.36)	90 (26.62)	73 (21.59)	<0.001 ^*∗*^
8–10	84 (24.85)	25 (7.39)	32 (9.46)	27 (7.98)	
More than 10	83 (24.55)	70 (20.71)	11 (3.25)	2 (0.59)	

^*∗*^Chi-square test.

## Data Availability

The data that support the findings of this study are available from the corresponding author upon reasonable request.
